# The institutional learning curve is associated with survival outcomes of robotic radical hysterectomy for early-stage cervical cancer-a retrospective study

**DOI:** 10.1186/s12885-020-6660-7

**Published:** 2020-02-24

**Authors:** Kyung Jin Eoh, Jung-Yun Lee, Eun Ji Nam, Sunghoon Kim, Sang Wun Kim, Young Tae Kim

**Affiliations:** 1 Department of Obstetrics and Gynecology, Yonsei University College of Medicine, Yongin Severance Hospital, Yongin, Gyeonggi-do 446-916 South Korea; 2 Division of Gynecologic Oncology, Department of Obstetrics and Gynecology, Severance Hospital, Institute of Women’s Life Medical Science, Yonsei University College of Medicine, 50-1 Yonsei-ro, Seodaemun-gu, Seoul, 03722 South Korea

**Keywords:** Learning curve, Hysterectomy, Cervical cancer

## Abstract

**Background:**

Despite recent advances in diagnosis and treatment, cervical cancer continues to be a significant health problem worldwide. Whereas robot-assisted surgery has advantages over the abdominal approach, and minimally invasive techniques are being used increasingly, these may be associated with a higher recurrence rate and lower overall survival than the abdominal approach. The objective of this study was to compare the surgical and survival outcomes between abdominal radical hysterectomy (ARH) and robotic radical hysterectomy (RRH).

**Methods:**

A retrospective cohort of patients undergoing radical hysterectomy for cervical cancer from 2006 to 2018 was identified. Patients with stage IA to IB cervical cancer were included and grouped: ARH vs. RRH. The RRH group was further divided into two groups based on the year of enrollment: RRH1 (2006–2012) and RRH2 (2013–2018). Tumor characteristics, recurrence rate, progression-free survival (PFS), and overall survival (OS) were compared between the groups. *P*-values < 0.05 (two-sided) were considered statistically significant.

**Results:**

A total of 310 patients were identified: 142 and 168 underwent ARH and RRH, respectively. RRH1 and RRH2 had 77 and 91 patients, respectively. Interestingly, RRH2 was more likely to have a larger tumor size (1.7 ± 1.4 vs. 2.0 ± 1.1 vs. 2.4 ± 1.7 cm, *P* = 0.014) and higher stage (*P* < 0.001) than RRH1. However, RRH2 showed significantly favorable PFS in contrast to RRH1. There was no difference between ARH and RRH2 in PFS (*P* = 0.629), whereas overall, the RRH group showed significantly shorter PFS than the ARH group. In the multivariate analysis, the institutional learning curve represented by the operation year was one of the significant predictors for PFS (hazard ratio [HR] 0.065, *P* = 0.0162), along with tumor size (HR 5.651, *P* = 0.0241).

**Conclusions:**

The institutional learning curve, represented by the operation year, is one of the most significant factors associated with outcomes of RRH for early-stage cervical cancer.

## Background

Although the recent widespread implementation of screening and prevention has decreased the incidence and mortality rates of cervical cancer, it continues to be a major public health problem [1]. Patients with early-stage cervical cancer are universally regarded as being ideal candidates for radical hysterectomy and pelvic lymph node (LN) dissection [2].

Conventionally, only the abdominal approach has been performed, but as technology related to minimally invasive surgery (MIS) continues to develop, the mainstream approach has been shifting to laparoscopic and robot-assisted surgery in radical hysterectomy [3, 4]. Further, previous studies have shown that the robot-assisted approach has several advantages over the abdominal approach, including decreased blood loss, higher counts of harvested LNs, fewer major complications, and shorter hospital stay [5–12].

However, recently released data from the Laparoscopic Approach to Cervical Cancer (LACC) trial (NCT00614211) indicated a higher recurrence rate and lower overall survival (OS) in patients with cervical cancer who were surgically treated with MIS than in those treated via the abdominal approach [13]. However, the unfavorable outcome of the MIS arm in the LACC trial could be a result of the surgical technique or negligence of the surgeon, rather than due to the MIS itself.

The aim of our study was to compare patient features, tumor characteristics, and survival outcomes in a retrospective cohort of patients who underwent abdominal radical hysterectomy (ARH) versus robotic radical hysterectomy (RRH) for cervical cancer at a tertiary referral institution and to evaluate factors that could impact the oncologic outcomes of RRH.

## Methods

### Patients

A retrospective cohort of patients who underwent RRH or ARH for cervical cancer between 2006 and 2018 at Yonsei Cancer Center, Severance Hospital was identified. Clinical data, including patient demographics, tumor characteristics, and clinical outcomes, were abstracted from the electronic medical records. All patients with a preoperative diagnosis of cervical cancer of squamous cell, adenocarcinoma, or adenosquamous histologies with a Federation of Gynecology and Obstetrics (FIGO) stage (prior to the revision in 2018) of less than II were included [14]. Those who received neoadjuvant chemotherapy prior to the surgery, whose FIGO stage was II, or who had histologies other than squamous cell, adenocarcinoma, or adenosquamous were excluded. Progression-free survival (PFS) was defined as the time interval between surgery and the first evidence of any recurrence or last follow-up. OS was described as the duration of time from the date of diagnosis to the date of death or last follow-up. The study was approved by the Institutional Review Board at Yonsei University College of Medicine.

### Surgical techniques

The type of surgical approach was determined after a discussion with each patient about the risks and benefits of both options. All patients in this cohort underwent type B-to-C radical hysterectomy, as described by Querleu and Morrow [15]. A systematic pelvic lymphadenectomy was performed, which included removal of the internal iliac nodes, external iliac nodes, obturator nodes, and common iliac nodes. Since the introduction of sentinel LN (SLN) biopsy in surgery for cervical cancer, it has been performed in our institution at the discretion of the surgeon [16]. All the radical hysterectomies were performed by the same board-certified gynecologic oncologists at a single tertiary referral hospital and assisted by gynecologic oncology fellows.

### Statistical analysis

Differences in patient demographics and tumor characteristics were compared using the Student’s t test and Chi-square test where appropriate. Cox proportional hazards regression analysis was used to estimate hazard ratios (HRs) and 95% confidence intervals (CIs). The Kaplan-Meier analysis was used to estimate the change in survival. *P*-values < 0.05 (two-sided) were considered statistically significant. Numerical data are presented as number (%) or the median ± standard deviation. Statistical analyses were performed using SPSS version 25.0 for Windows (IBM, Chicago, IL, USA) and R Statistical Software version 3.6.1 (Foundation for Statistical Computing, Vienna, Austria).

## Results

**Fig. 1:**
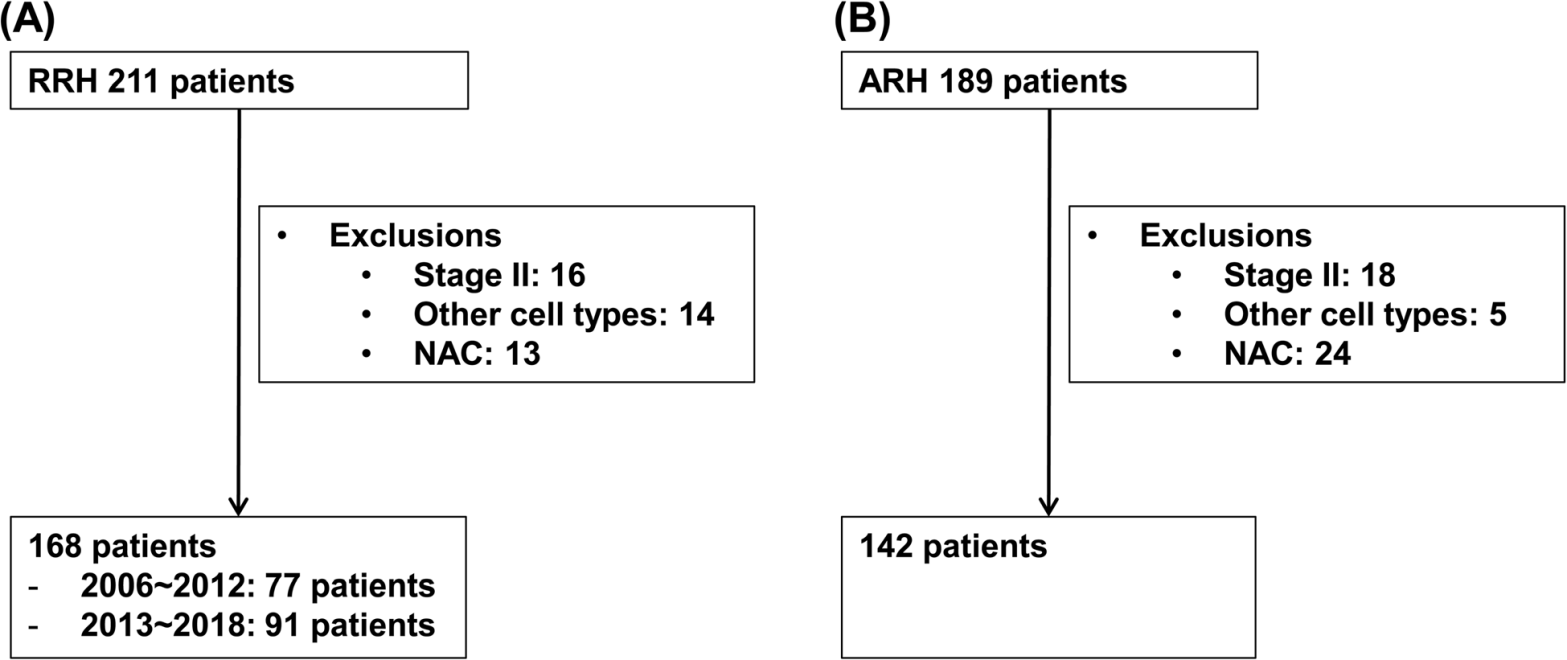
Flowchart of patient selection. Selection of patients who underwent RRH (**a**) and ARH (**b**). RRH, robotic radical hysterectomy; ARH, abdominal radical hysterectomy; NAC, neoadjuvant chemotherapy

Figure 1 shows a flowchart of the patient selection process. In total, 310 patients were identified, of whom 142 underwent ARH and 168 underwent RRH. Patients who underwent RRH between 2006 and 2012 were classified as RRH1, and patients who underwent RRH between 2013 and 2018 were classified as RRH2. RRH1 and RRH2 consisted of 77 and 91 patients, respectively.

Patients in RRH2 were more likely to have a higher stage, compared with ARH or RRH1 (*P* < 0.001). The ARH group was significantly younger than the two other groups (*P* = 0.002). Body mass index and cell type were not significantly different among the three groups (Table 1). Table 2 presents the outcomes of surgery and postoperative adjuvant treatments. The ARH group showed deeper invasiveness (*P* < 0.001) and more lymphovascular space invasion (LVSI; *P* < 0.001). In RRH2, a significantly reduced number of harvested LNs was observed, which is expected to be the result of the SLN biopsy introduced in our hospital in 2012. Additionally, RRH2 was more likely to have a larger tumor size than the ARH group (2.4 ± 1.7 vs. 1.7 ± 1.4 cm, *P* = 0.014).

**Table 1:** Patient characteristics

	ARH (*N* = 142)	RRH1 (‘06–'12) (*N* = 77)	RRH2 (‘13–'18) (*N* = 91)	*P*
Age	49.7 ± 11.3	46.7 ± 10.3	45.3 ± 9.8	0.002
BMI	23.5 ± 3.4	22.8 ± 4.0	23.4 ± 3.2	0.761
Stage				< 0.001
1A1	14 (9.9%)	12 (15.6%)	12 (13.2%)	
1A2	10 (7.0%)	2 (2.6%)	1 (1.1%)	
1B1	90 (63.4%)	63 (81.8%)	68 (74.7%)	
1B2	28 (19.7%)	0	10 (11.0%)	
Cell type				0.157
SCC	106 (74.6%)	58 (75.3%)	60 (65.9%)	
AC	32 (22.5%)	18 (23.4%)	31 (34.1%)	
AS	4 (2.8%)	1 (1.3%)	0	

*ARH* Abdominal radical hysterectomy, *RRH* Robotic radical hysterectomy, *BMI* Body mass index, *SCC* Squamous cell carcinoma, *AC* Adenocarcinoma, AS Adenosquamous

**Table 2:** Pathological results and postoperative treatment

	ARH (*N* = 155)	RRH1 ('06–'12) (N = 77)	RRH2 ('13–'18) (N = 91)	*P*
Depth of invasion	0.8 ± 0.5	0.6 ± 0.6	0.5 ± 0.5	< 0.001
LVSI	55 (50.5%)	21 (27.3%)	19 (20.9%)	< 0.001
Harvested pelvic LNs	19.7 ± 9.8	16.6 ± 9.2	9.6 ± 9.0	< 0.001
LN metastasis	16 (11.3%)	4 (5.2%)	9 (9.9%)	0.188
Tumor size	1.7 ± 1.4	2.0 ± 1.1	2.4 ± 1.7	0.014
Postoperative treatment				0.001
RT	28 (19.7%)	5 (6.5%)	4 (4.4%)	
POAC	9 (6.3%)	6 (7.8%)	1 (1.1%)	
CCRT	9 (6.3%)	7 (9.1%)	11 (12.1%)	

*ARH* Abdominal radical hysterectomy, *RRH* Robotic radical hysterectomy, *LVSI* Lymphovascular space invasion, *LN* Lymph node, *RT* Radiotherapy, *POAC* Postoperative adjuvant chemotherapy, *CCRT* Concurrent chemoradiotherapy

In the multivariate analysis, the institutional learning curve, represented by the year of operation, was one of the significant predictors for PFS (HR 0.065, *P* = 0.0162), along with tumor size (HR 5.651, *P* = 0.0241) (Table 3). Moreover, LVSI and postoperative treatments were also observed to be possible predictors of PFS but did not reach statistical significance.

**Table 3:** Multivariate analysis of various factors correlated with progression-free survival

	No. of patients	PFS	
		Multivariate analysis	
		HR (95% CI)	*P*
Age, years (continuous)	168	1.025 (0.956–1.099)	0.4894
LC			
2006~2012	77	1 (Reference)	
2013~2018	91	0.065 (0.007–0.603)	0.0162*
BMI			
< 25	130	1 (Reference)	
≥ 25	38	0.243 (0.025–2.404)	0.2264
Stage			
IB1, IB2	141	1 (Reference)	
IA1, IA2	27	0.638 (0.092–4.446)	0.6502
Histology			
SCC	118	1 (Reference)	
AC & AS	50	1.367 (0.375–4.986)	0.6357
Invasiveness			
< 0.3 cm	97	1 (Reference)	
≥ 0.3 cm	71	1.139 (0.297–4.363)	0.8493
LVSI			
No	128	1 (Reference)	
Yes	40	4.590 (0.973–21.659)	0.0543
No. of harvested LNs			
< 20	129	1 (Reference)	
≥ 20	39	0.325 (0.068–1.557)	0.1597
Metastatic LNs			
No	155	1 (Reference)	
Yes	13	2.609 (0.424–16.041)	0.3006
Tumor size			
< 2 cm	125	1 (Reference)	
≥ 2 cm	43	5.651 (1.255–25.448)	0.0241*
Postoperative treatment			
Yes	34	1 (Reference)	0.0755
No	134	0.185 (0.029–1.189)	

*PFS* Progression-free survival, *HR* Hazard ratio, *CI* Confidential interval, *LC* Learning curve, *BMI* Body mass index, *SCC* Squamous cell carcinoma, *AC* Adenocarcinoma, *AS* Adenosquamous, *LVSI* Lymphovascular space invasion, *LN* Lymph node; *, *P* < 0.05

**Fig. 2:**
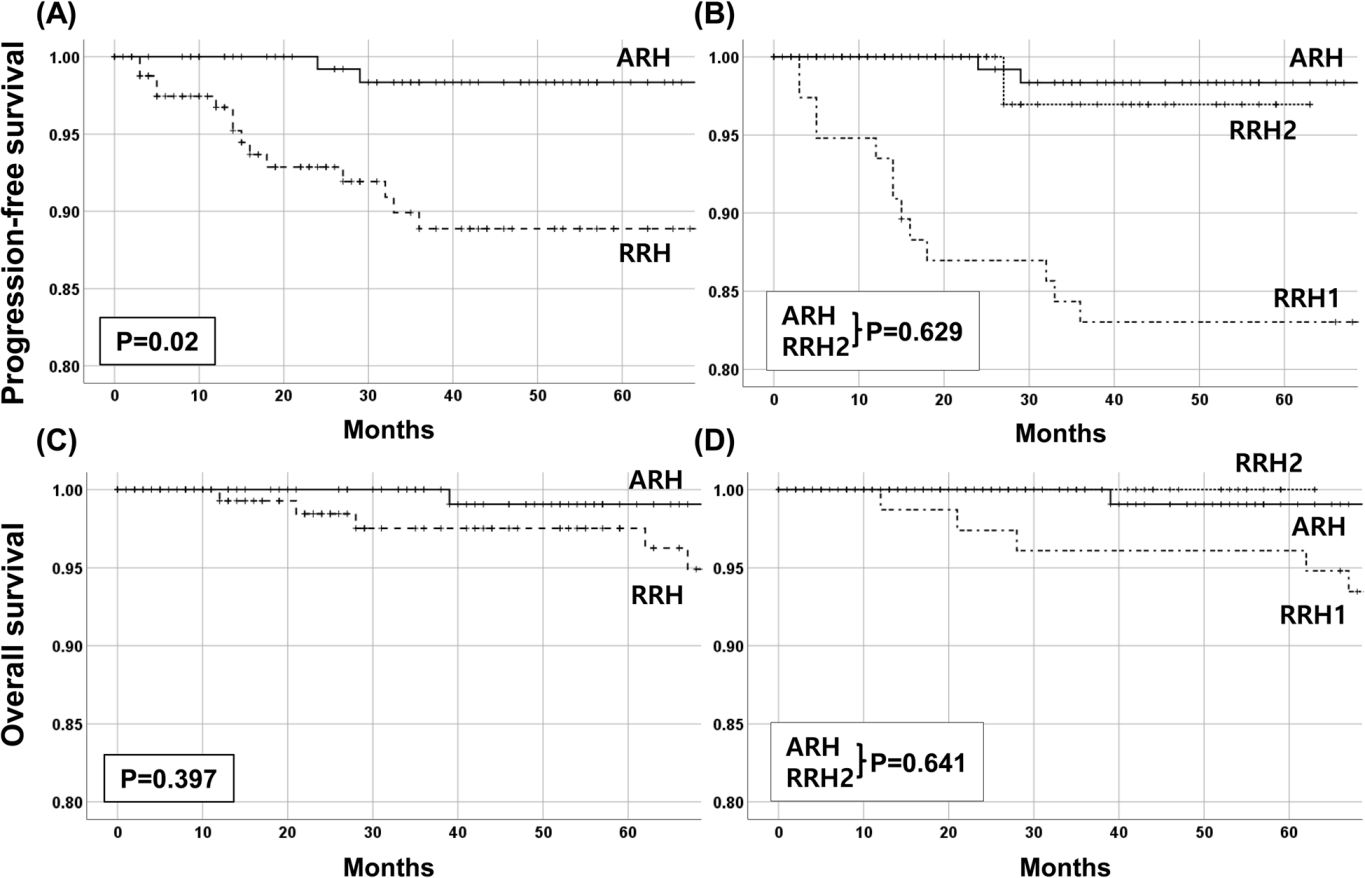
Survival analysis. Comparison of progression-free survival (**a**, **b**) and overall survival (**c**, **d**) in ARH vs. RRH (**a**, **c**) and ARH vs. RRH1 vs. RRH2 (**b**, **c**). RRH, robotic radical hysterectomy; ARH, abdominal radical hysterectomy

PFS was significantly different between the ARH group and the overall RRH group (*P* = 0.002), but there was no difference between the ARH group and RRH2 (*P* = 0.629; Fig. 2a, b). OS did not differ significantly between ARH and RRH, and there was no significant difference among ARH, RRH1, and RRH2 (Fig. 2c, d).

## Discussion

In this study, we compared the surgical outcomes of ARH and RRH for cervical cancer. In particular, RRH was analyzed by dividing the cohort according to the year of surgery into the first half (RRH1) and the latter half (RRH2). Interestingly, classification according to the year of performance, which is thought to reflect the institutional learning curve, was found to be a significant PFS predictor along with known factors such as tumor size.

Previous retrospective studies have indicated that there is no survival difference between robot-assisted and abdominal approaches, which is consistent with our results [17–21]. In addition, even when stratified by tumor size, oncologic outcomes were not significantly different between laparoscopic and abdominal approaches, which may emphasize the importance of the learning curve over the mode of surgery itself [22, 23]. In this study, by changing the viewpoint, a comparative analysis was performed using the year of surgery to reflect the learning curve of the institution as an independent factor, which was shown to be the most significant predicting factor for oncologic prognosis.

The results of the LACC trial, a multi-center randomized phase III trial evaluating the long-term survival of women who underwent minimally invasive radical hysterectomy vs. ARH, were presented at the 2018 Society of Gynecologic Oncology (SGO) annual meeting [13]. This study included patients with stages IA1 with LVSI, IA2, and IB1 disease and randomized 631 patients to radical hysterectomy using MIS or abdominal approaches. The LACC trial was indecisive with respect to its primary objective of disease-free survival as the CI crossed the predetermined noninferiority margin of − 7.2 percentage points for MIS (difference, − 10.6 percentage points; 95% CI − 16.4 to − 4.7, *P* = 0.87 for noninferiority). However, the secondary endpoints of disease-free survival and OS favored the open surgery group. The MIS RH surgery group showed a significantly lower 3-year disease-free survival and OS rate than the open RH surgery group (3-year rate, 91.2% vs. 97.1%; HR for disease recurrence or death from cervical cancer, 3.74; 95% CI, 1.63 to 8.58). These unexpected results have already led to a change in practice patterns at many institutions, which now have completely terminated or significantly reduced the application of MIS for cervical cancer based on the results of this trial.

In addition, the results of the LACC trial were consistent with those of a retrospective analysis using the Surveillance, Epidemiology, and End Results data of the National Cancer Institute in the USA, which argues that the introduction of MIS was associated with an increased mortality rate due to cervical cancer [24]. In the analysis, MIS was associated with an increased probability of mortality within 4 years compared to laparotomy (9.1% vs. 5.3%). Nonetheless, for patients who had tumors < 2 cm, the HR for death was statistically similar between the two surgical approaches in the subgroup analysis. Other retrospective studies concluded that MIS was associated with decreased survival in women who had tumors ≥2 cm [25, 26].

Certain points in the LACC trial, however, have faced criticism. The LACC trial design included surgeons who could submit data from only 10 MIS cases and 2 unedited videos, to exclude the contributing centers’ learning curve. However, many gynecologic oncologists suspect that this could not sufficiently support evidence that properly trained surgeons contributed in the MIS arm [27]. Also, we should focus on the result that only 7 recurrences (2.2%) were observed in the 312 women in the open surgery arm, which is an extremely low rate of recurrence comparing with previous reports, whereas 27 (8.4%) recurrences were noted in the MIS arm, which is comparative to the data reported in previous studies [18, 19, 23, 28, 29]. This observation suggested that the surgeons who already had overcome the learning curve for MIS; therefore, adopting the MIS approach for cervical cancer as the first option might have been excluded in the LACC trial at the beginning. Moreover, despite including a combination of both conventional laparoscopy and robotic surgery in the MIS arm, the enrolment was heavily skewed toward laparoscopy, and only 15.6% (*N* = 45) of women had undergone robotic hysterectomy. Additionally, a substantial proportion of data was missing, with unknown grade (29%) or depth of invasion (33%). Therefore, a well-controlled study that addresses all the above-mentioned concerns is required.

The strengths of this study were that it was conducted at a single tertiary referral institution performing high-volume robot-assisted surgery for cervical cancer and that it compared robotic surgery alone with ARH. Additionally, the characteristics of the included patient population were similar to those of patients included in the LACC trial. Thus, the concerns raised by the relatively small number of RRHs in the LACC trial can be addressed. However, there are several limitations related to the retrospective design of this study, including the potential for selection bias, unmeasured confounders, and missing data that may have affected data analysis.

## Conclusions

Our study found that institutional experience with robotic surgery, represented by the operation year, is one of the most significant factors associated with RRH outcomes for early-stage cervical cancer. We should not discard all the benefits of robot-assisted laparoscopy by doing away with the minimally invasive approach for cervical cancer. Before the well-controlled trial is carried out, the mode of surgery should be determined according to each surgeon’s proficiency. Surgeons are recommended to counsel their patients and decide on the mode of surgery based on the oncologic outcomes of the previous institutional patients.

## Data Availability

The datasets used and/or analyzed during the current study are available from the corresponding author on reasonable request.

## Abbreviations

AC: Adenocarcinoma
ARH: Abdominal radical hysterectomy
AS: Adenosquamous
BMI: Body mass index
CCRT: Concurrent chemoradiotherapy
LN: Lymph node
LVSI: Lymphovascular space invasion
POAC: Postoperative adjuvant chemotherapy
RRH: Robotic radical hysterectomy
RT: Radiotherapy
SCC: Squamous cell carcinoma
